# Anaesthetic Management of A Case of Osteogenesis Imperfecta with Urinary Bladder Stone - A Case Report

**Published:** 2009-02

**Authors:** Munish Garg, Manish Jain, Amit Gupta

**Affiliations:** 1 Assistant Prof, Department of Anesthesiology & Critical Care, Subharti Medical College, Meerut, U.P., INDIA; 2 Associate Prof, Department of Anesthesiology & Critical Care, Subharti Medical College, Meerut, U.P., INDIA; 3 Assistant Prof, Department of Anesthesiology & Critical Care, Subharti Medical College, Meerut, U.P., INDIA

**Keywords:** Osteogenesis Imperfecta, Bladder stone, Anaesthesia

## Abstract

**Summary:**

Sometimes in practice of anaesthesia, anaesthesiologist encounters patients with rare congenital diseases. To anaesthesiologist, these patients are a challenge due to inherent complications associated with the disease. Here, we are reporting a case of osteogenesis imperfecta who was posted for the surgery for vesical calculus. All investigations were done to rule out any cardio-respiratory abnormalities, bleeding disorders, which are commonly associated with these patients. Caudal epidural was chosen as anaesthesia technique of choice as spinal anaesthesia was anticipated to be difficult due to associated kyphoscoliosis. GA was avoided due to anticipated difficult airway, restrictive lung disease and susceptibility to malignant hyperthermia. We emphasize the importance of proper preanaesthetic evaluation, intellectual, mental and logistical preparation which should be done before anaesthetising these types of patients.

## Introduction

Osteogenesis imperfect (OI), also known as brittle bone disease, is agenetic disorder of connective tissue characterized by bones that fracture easily, often with little or no trauma. Osteogenesis imperfecta is caused by a faulty gene that instruct to make too little or poor quality of type 1 collagen^1^. The prevalence of osteogenesis imperfecta ranges from 1:60000 to 1:20000^2^ depending upon type of OI. Inheritance in nearly all cases follows an autosomal dominance pattern, although sporadic cases are common. The disorder is frequently associated with blue sclera, dental abnormalities^3^ (dentinogenesis imperfecta), progressive hearing loss, and a positive family history. The most common classification for OI was developed by Sillence^4^.

Anaesthetic implication of OI includes difficult intubation^5^, platelet dysfunction, cardiovascular abnormalities like mitral valve prolapse^6^,^7^, tendency to develop malignant hyperthermia^8^,^9^ and problems with positioning of patient due to brittle bones.

## Case report

**Fig 1:**
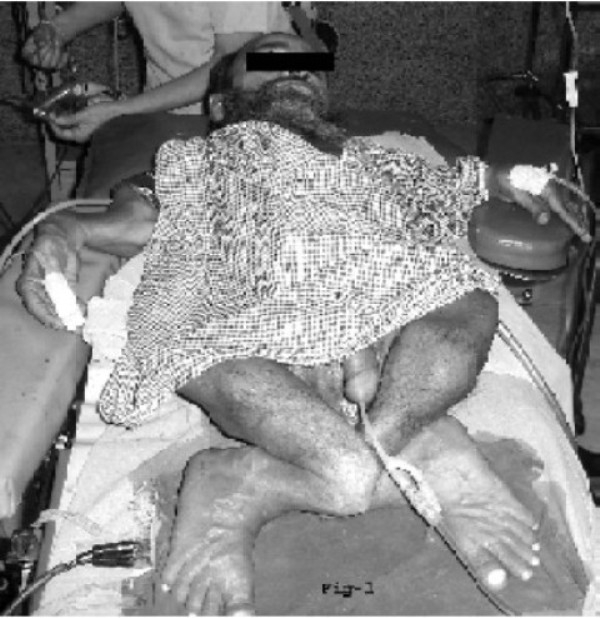
Photograph showing skeletal abnormalities

**Fig 2:**
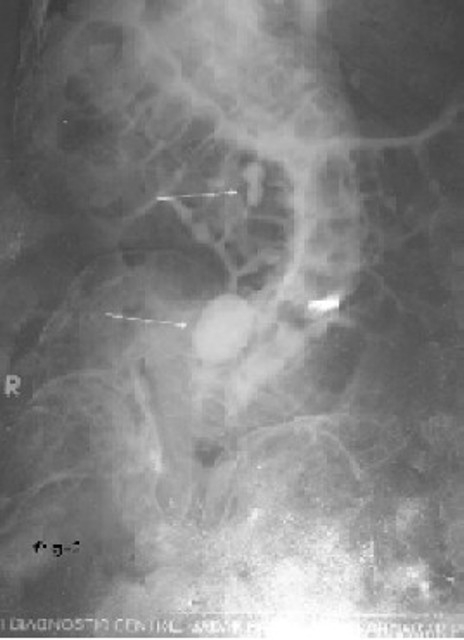
X Ray showing urinary bladder and renal stone

A 54-year-old male patient presented in emergency room with acute retention of urine. He was a known case of OI tarda (Fig 1). Immediately foley's catheterization (no.-16fr.) was done to relieve retention. X-Ray KUB of the patient revealed a right side renal calculus and a vesicle calculus and was planned for cystolithotomy (Fig 2).

On preanaesthetic evaluation, the patient weighed 22 kg, length 94 cm, head circumference 50 cm, chest circumference 65 cm, and was bedridden. He had undergone previously cystolithotomy at 6 months of age (details of that operation were not available). History revealed uneventful antenatal period and no delay in milestones. Patient developed normally upto 10yr of age and after that he started having skeletal fractures. There was no positive family history.

Investigation revealed Hb 12.6 g%, TLC 9300, platelet count 2.3 lacs, blood sugar 86 mg/dl, blood urea 40.4 mg/dl, serum creatinine 0.6 mg/dl, serum potassium 4.5meq/l, serum sodium 143meq/l, serum calcium 8.7meq/l.

Preoperative coagulation profile was normal. 2D echo revealed no valvular abnormalities and normal ejection fraction (64%). Pulmonary function tests revealed a moderate degree of restrictive lung disease.

The patient was accepted for surgery as ASA grade II. In view of anticipated difficult airway, it was planned to conduct the case under regional anaesthesia. In the operation theatre, i.v. line was secured using 18 G cannula. Monitoring included 5 lead ECG, manual blood pressure monitoring, SpO2, EtCO2, and temperature. Difficult airway cart was also kept standby. After preloading with 500ml of Ringer lactate i.v., caudal epidural anaesthesia was given in lateral position with 14ml of 0.25% of bupivacaine. Midazolam 1.0mg and butorphanol 1.0mg i.v. was given for sedation. During surgery, his vitals remained stable and no rescue medication was required. Operation lasted for about 40 min and patient had an uneventful recovery. He was discharged on 5^th^ postoperative day.

## Discussion

Osteogenesis imperfecta is a rare autosomal dominant inherited disease of connective tissues that affects bones, sclera and inner ear^10^. The incidence is higher in females. Clinically, it occurs in two forms: osteogenesis imperfecta congenita and osteogenic imperfecta tarda. With congenital forms fractures occur in utero and death is usually in perinatal period. The tarda form typically manifests during childhood or early adolescence, but the patients have a normal lifespan.

Management of anaesthesia is influenced by coexisting orthopaedic deformities^11^, vulnerabilities to fracture during perioperative period, associated cardiac abnormities, impaired platelet function, tendency to develop hyperthermia and rarely extra skeletal manifestations^12^. Due to abnormal skeletal growth difficult airway must always be anticipated in such patients. Associated kyphoscoliosis along with pectus carinatum may decrease vital capacity, chest wall compliance with resulting arterial hypoxemia due to ventilation perfusion mismatch and this can lead to increased risk under GA. Succinylcholine should be avoided as fasciculation can lead to fractures. Regional anaesthesia is acceptable in selected patients as it avoids need for tracheal intubation but may be difficult because of kyphoscoliosis. Before giving regional anaesthesia, coagulation profile must be screened due to associated increase in bleeding time despite normal platelet count^13^.

For monitoring of blood pressure, automated blood pressure cuffs may be hazardous as over inflation may result in fracture. During prolonged surgery, all pressure points should be well padded and positioning of patient along with transportation^14^ should be very gentle to prevent occurrence of fracture.

There have been several successful case reports of conductance of surgery under general anaesthesia in patients with osteogenesis imperfecta. Karabiyik et al^1^ have recommended TIVA along with ILMA to manage elective case, while Malde et al^14^ have successfully used balanced general anaesthesia in a case of osteogenesis imperfecta with gross deformity of pelvis for abdominal hysterectomy.

In our patient, we avoided general anaesthesia due to anticipated difficult airway (attributed to limited mobility of cervical spine, short neck and absent dentition), restrictive lung disease (due to associated kyphoscliosis, pectuscarinatum) and susceptibility to malignant hyperthermia. However, preparation was kept ready in operation theatre for managing difficult airway in case of emergency.

We preferred regional anaesthesia since the patient had to undergo a lower abdominal surgery and to avoid risk related to general anaesthesia. Before giving regional anaesthesia a thorough preoperative workup of patient was done with special attention to coagulation profile as these patients are prone to have abnormal bleeding tendencies. Patient's BT, CT and PT were within normal limits. Caudal epidural was chosen as preferred anaesthesia technique over spinal anaesthesia as it was difficult to perform lumbar puncture due to associated kyphoscoliosis and unpredictability of the level of block. The effect of caudal block was till T10 level and course of surgery was uneventful.

To summarise, patients with OI pose a significant challenge to anaesthesiologist owing to difficult airway, problems with positioning, fractures, tendency for hyperthermia and platelet functional abnormalities. Only thorough preoperative workup and prompt management can improve the outcome in these patients.
